# Supplementary material for: CdGAP promotes prostate cancer metastasis by regulating epithelial-to-mesenchymal transition, cell cycle progression, and apoptosis
Source: Commun Biol. 2021 Sep 7;4:1042. doi: 10.1038/s42003-021-02520-4 (PMC8423782; doi:10.1038/s42003-021-02520-4)
Supplement: Supplementary file 2 — Supplementary Information [file 42003_2021_2520_MOESM2_ESM.pdf]

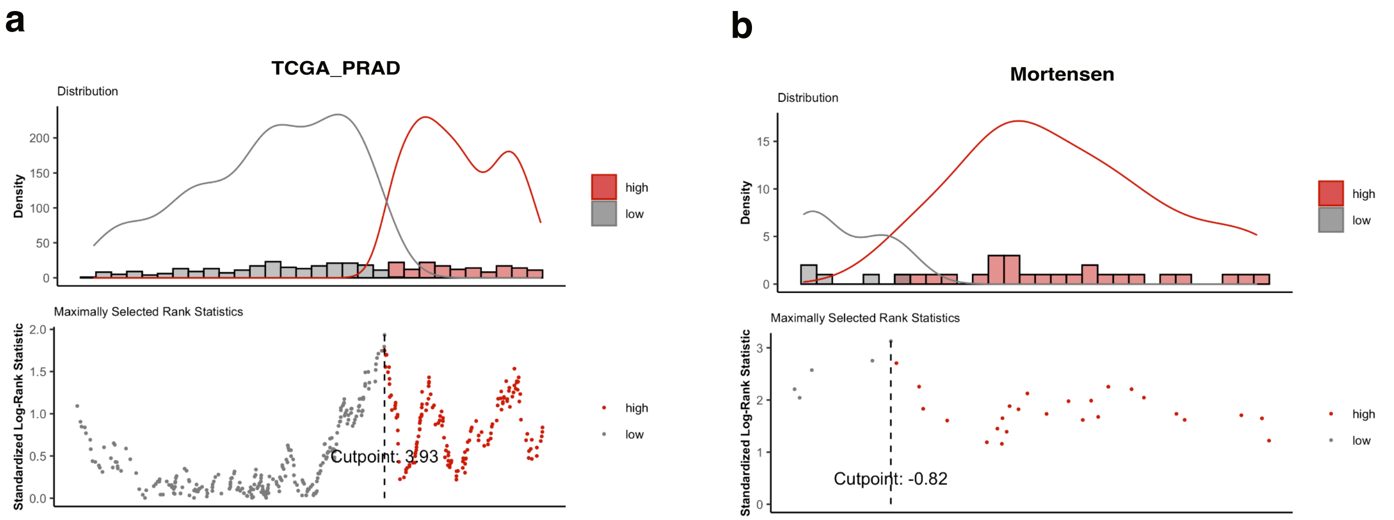

Supplementary Figure 1. a, b Dichotomization of patients in the TCGA_PRAD **(a)** and Mortensen datasets **(b)** divided into high expression and low expression groups by optimal cutpoint.

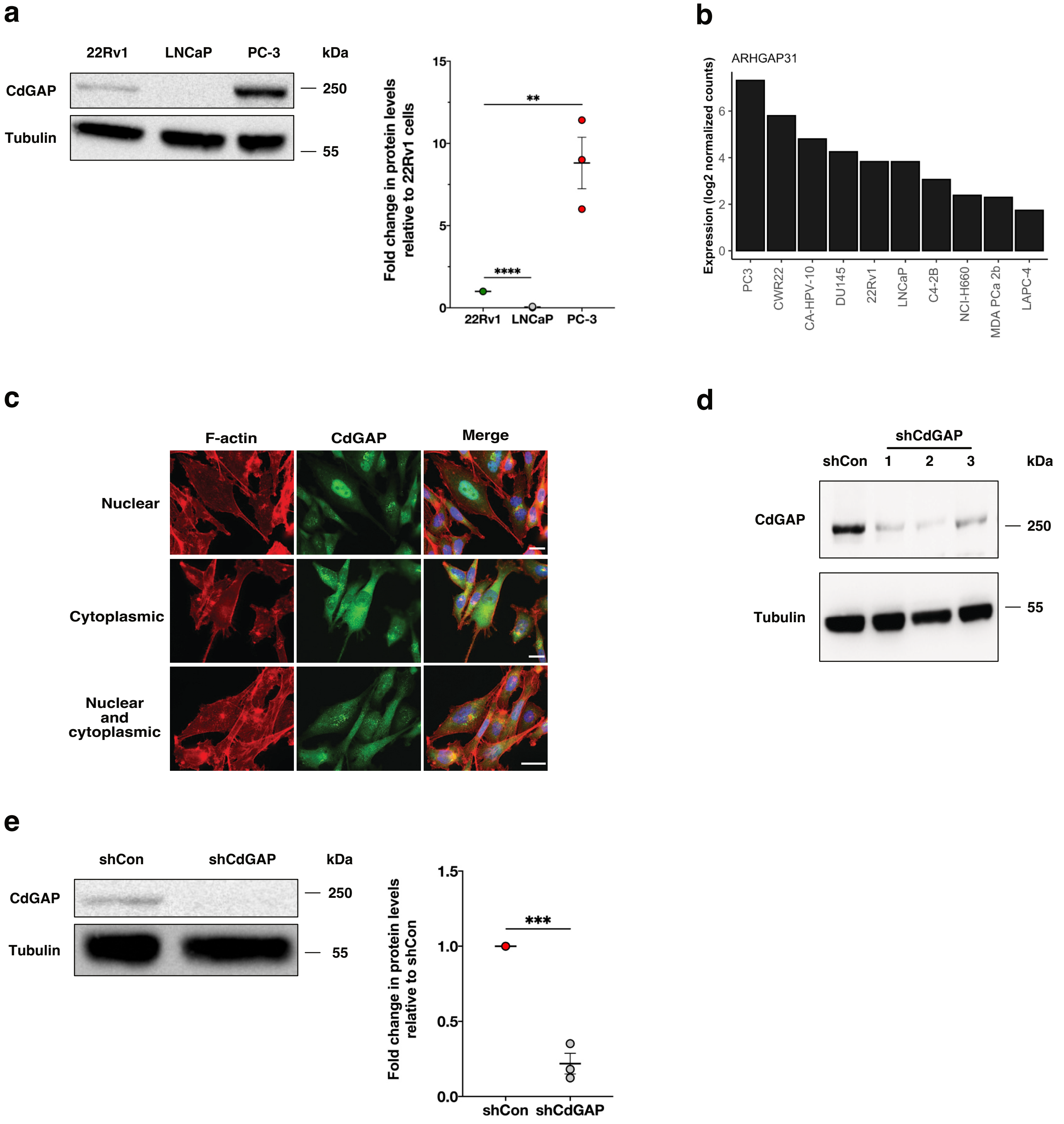

Supplementary Figure 2. **a** Immunoblot analysis of CdGAP in human prostate cancer cell lines 22Rv1, LNCaP and PC-3. Tubulin was used as a loading control. Graphs provide a densitometry analysis of CdGAP protein levels relative to 22Rv1 cells (n=3). **b** *ARHGAP31* transcript abundance in prostate cancer cell lines from the Prensner RNA-seq dataset. **c** PC-3 cells were fixed and then stained for CdGAP (green) or F-actin (red) with phalloidin. 4′,6′-diamidino-2-phenylindole (DAPI) was used to stain the nuclei. CdGAP nuclear (top panels), cytoplasmic (middle panels), or both (bottom panels) localization is represented. Scale bar represents 10 µm. **d** Immunoblot analysis of CdGAP levels in PC-3 cells infected with scrambled control (shCon) or shRNA targeting CdGAP (shCdGAP) single clones. Tubulin was used as loading control. **e** Immunoblot analysis of CdGAP levels in 22Rv1 cells infected with scrambled control (shCon) or shRNA targeting CdGAP (shCdGAP). Tubulin was used as loading control. Graphs provide a densitometry analysis of CdGAP protein levels in CdGAP-depleted cells relative to shCon (n=3). Two-sample unpaired Student's t-test for comparison between two groups (shCon;shCdGAP). Error bars indicate SEM. ****, $p < 0.0001$ ***, $p < 0.001$; **, $p < 0.01$.

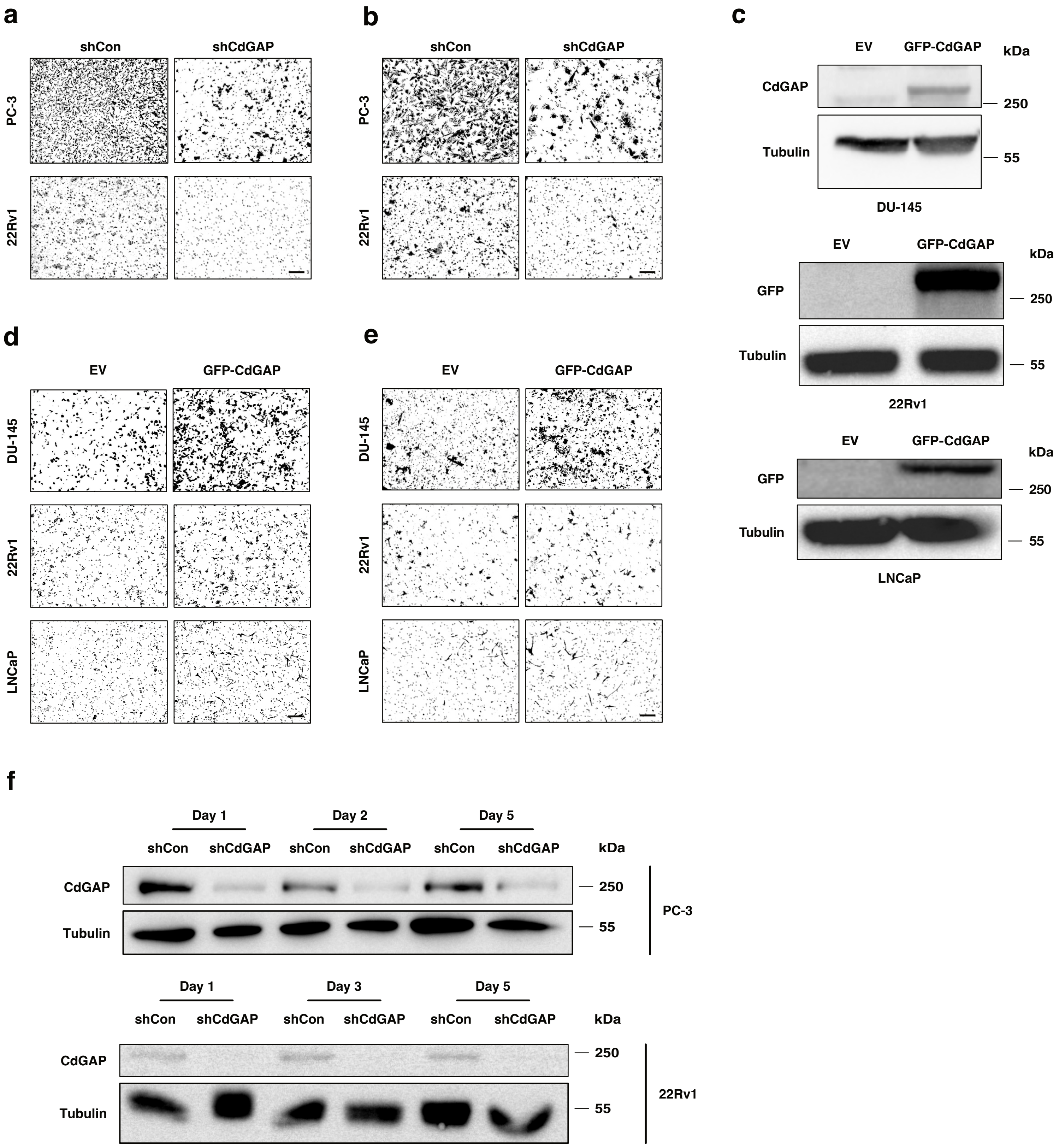

Supplementary Figure 3. a,b Representative images from transwell migration and invasion assays of CdGAP-depleted PC-3 and 22Rv1 cells with corresponding controls. Scale bar represents 100 µm. **c** Immunoblot analysis of CdGAP from protein lysates of DU-145, 22Rv1, and LNCaP cells transfected with pEGFP-C1 empty vector (EV) or pEGFP-CdGAP. Tubulin was used as loading control. **d,e** Representative images from transwell migration (d) and invasion (e) assays of DU-145, 22Rv1, and LNCaP cells transfected with either empty vector (EV) or GFP-CdGAP. Scale bar represents 100 µm. f Immunoblot analysis of CdGAP from protein lysates of CdGAP-depleted PC-3 and 22Rv1 cells with corresponding controls over a period of 5 days in culture.

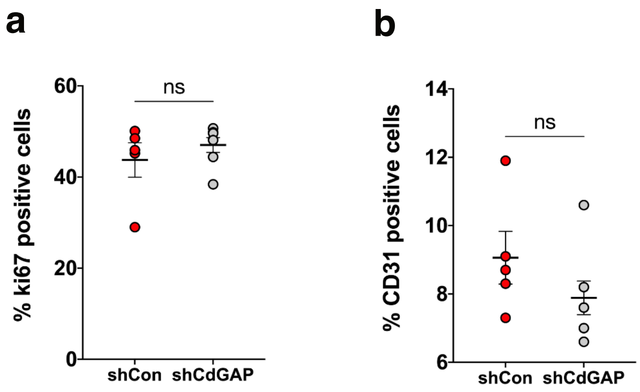

Supplementary Figure 4. a, b Quantification of Ki-67 (a) and CD-31 (b) positive cells by IHC staining in primary tumors from control (shCon) or CdGAP-depleted (shCdGAP) PC-3 cells-injected mice. Error bars indicate SEM. ns = not significant.

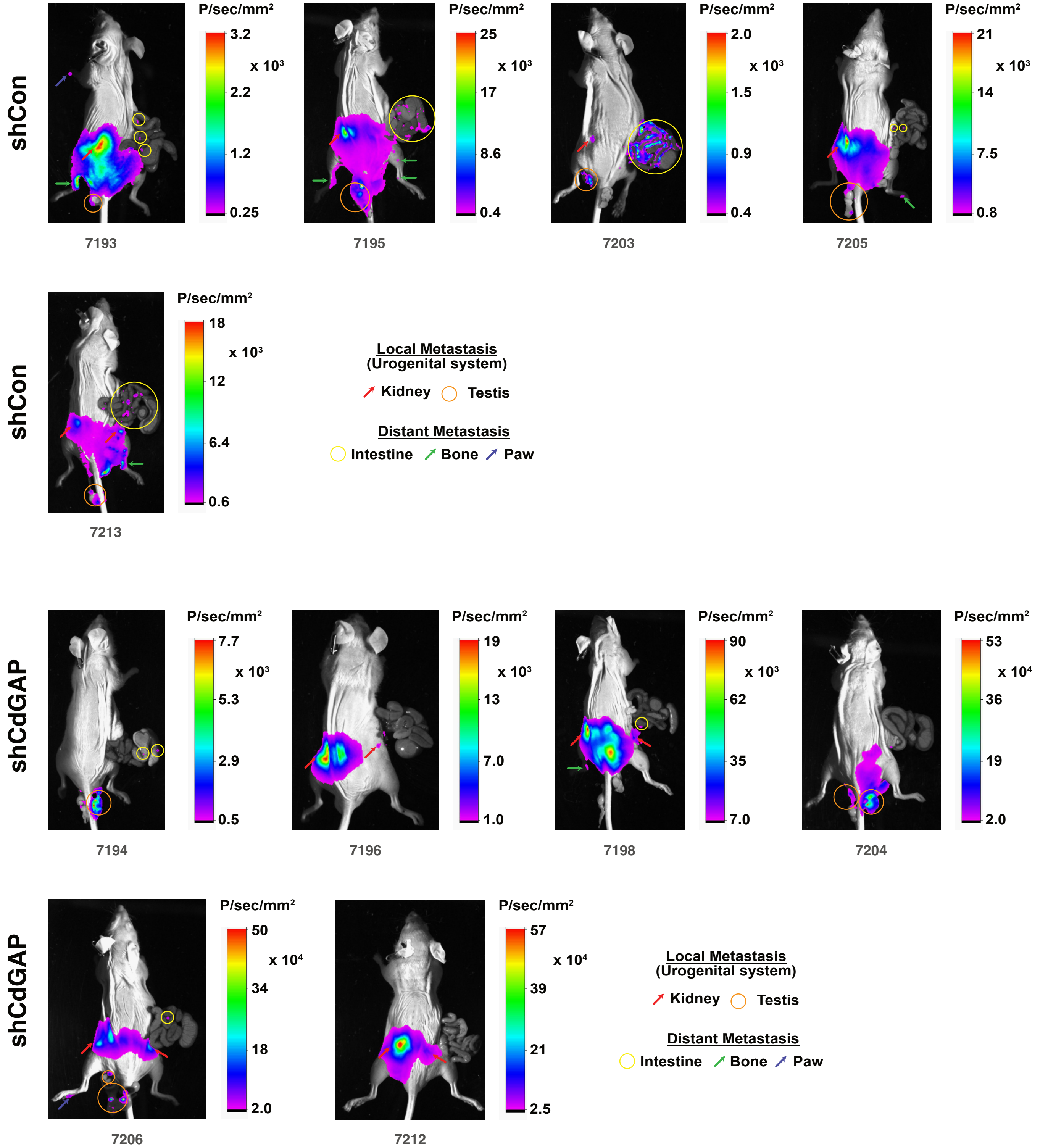

Supplementary Figure 5. Ex vivo imaging of mice injected with control (shCon) or CdGAP-depleted PC-3 (shCdGAP) cells. At the experimental end date (28 days), mice were euthanized, primary tumors were removed and then immediately subjected to ex vivo imaging performed on post-mortem mice to visualize metastasis. Each mouse was exposed for 4 minutes.

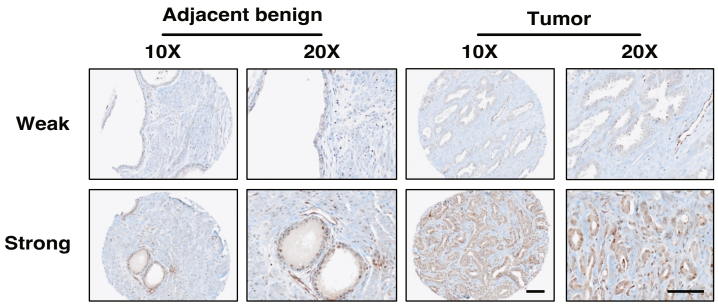

Supplementary Figure 6. Representative IHC of CdGAP expression on a panel of radical prostatectomy specimens from 285 prostate cancer patients using the TF123 TMA. Nuclear and cytoplasmic CdGAP staining was scored with an intensity varying from weak to strong (0 to 3) in adjacent benign and matched tumor tissue cores. Magnification, 10X and 20X. Scale bar represents 100 μm.

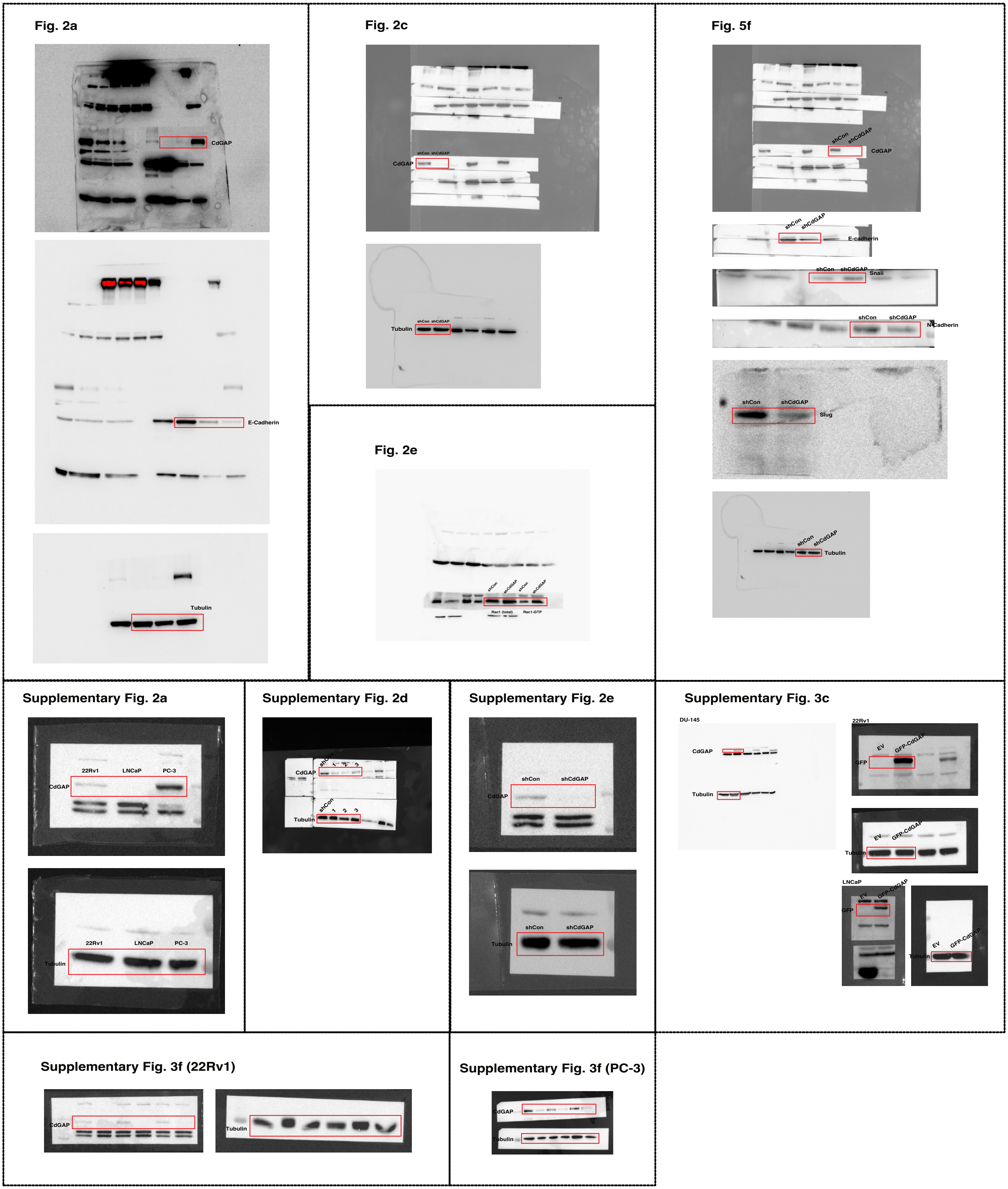

Supplementary Figure 7. Representative western blots for each antibody used in the manuscript.
Uncropped western blots.

Number of Patients		285	
Age at Diagnosis	Mean (years)	62	
Follow-up	Median (months)	143	
		N	%
Biochemical Relapse 60 months	No	190	67
	Yes	95	33
Bone Metastasis 10 years	No	265	93
	Yes	20	7
Castrate Resistant	No	259	91
	Yes	26	9
Death	Pca specific	20	7
	Other cause	45	16
	Overall	65	23
Gleason Score Radical Prostatectomy	≤3+3	139	49
	3+4	94	33
	4+3	19	7
	≥4+4	29	10
	U	4	1
pTNM	2	200	70
	3	76	27
	4	9	3
Lymph Node Invasion	No	196	69
	Yes	9	3
	U	80	28
Capsular Penetration	No	205	72
	Yes	80	28
Seminal Gland Invasion	No	269	94
	Yes	16	6
Margin status	Negative	185	65
	Positive	95	33
	U	5	2

Supplementary Table 1. Clinico-pathological information of the cohort of 285 prostate cancer patients participating in the Centre de Recherche du Centre Hospitalier de l'Université de Montréal prostate cancer biobank and included to the TF123 TMA.

	Parameter	BCR free survival (5 years)				Bone Metastasis free survival (10 years)			
		Univariate				Univariate			
		Sig.	HR	95.0% CI for HR		Sig.	HR	95.0% CI for HR	
				Lower	Upper			Lower	Upper
Clinical data	PSApreop	**<0.001**	1.059	1.032	1.087	0.055	1.055	0.999	1.115
	GleasonScoreCat1234	**<0.001**	1.837	1.537	2.195	**<0.001**	3.606	2.287	5.686
	Margin	**<0.001**	3.396	2.246	5.136	**0.022**	2.893	1.163	7.193
	pTNM	**<0.001**	2.735	2.027	3.688	**<0.001**	6.443	3.376	12.296
Continuous data	CdGAP Cytoplasm intensity in BA	0.292	0.624	0.259	1.501	0.714	0.685	0.09	5.192
	CdGAP Cytoplasm intensity in T	0.615	1.097	0.764	1.576	**0.005**	2.416	1.31	4.453

BA: adjacent benign; T: Tumor; **bold** p<0.05

Supplementary Table 2. Univariate analyses were used to estimate the hazard ratios (HRs) for CdGAP and other clinico-pathological parameters. The serum PSA levels prior to the radical prostatectomy, pathologic staging of the primary tumor (pT 2, 3, 4), Gleason Score category [6, 7 (3+4), 7 (4+3), 8+], and margin status (negative/positive) were included in the model.

Antibody name	Company: Catalog number	Dilution
CdGAP	Sigma: HPA036380	1 in 1,000
E-Cadherin	Cell Signaling: 3195	1 in 1,000
Snail 1	Cell Signaling: 3879	1 in 500
a -Tubulin	Sigma: T5168	1 in 1,000
Rac1	AbCAM: 23A8	1 in 1,000
N-Cadherin	BD Biosciences: 610920	1 in 1,000
Slug	Cell Signaling: C19G7	1 in 1,000
Anti-rabbit IgG	ThermoFisher: 45-000-682	1 in 10,000
Anti-mouse IgG	ThermoFisher: 45-000-679	1 in 10,000

Supplementary Table 3. List of antibodies used in this study.

Gene Name	Forward Primer	Reverse Primer
Snail1	CCCTCAAGATGCACATCCGAA	GACTCTTGGTGCTTGTGGAGCA
CDH1	CCCGCCTTATGATTCTCTGCTCGTG	TCCGTACATGTCAGCCAGCTTCTTG
P21	AGGTGGACCTGGAGACTCTCAG	TCCTCTTGGAGAAGATCAGCCG
N-Cadherin	CCTCCAGAGTTTACTGCCATGAC	GTAGGATCTCCGCCCTGATTC
Slug	TGTTGCAGTGAGGGCAAGAA	GAGCCTGGTTGCTTCAAGGA

Supplementary Table 4. List of primers used in this study.
