# Supplementary material for: CdGAP promotes prostate cancer metastasis by regulating epithelial-to-mesenchymal transition, cell cycle progression, and apoptosis
Source: Commun Biol. 2021 Sep 7;4:1042. doi: 10.1038/s42003-021-02520-4 (PMC8423782; doi:10.1038/s42003-021-02520-4)
Supplement: Supplementary file 3 — Description of Supplementary Files [file 42003_2021_2520_MOESM3_ESM.pdf]

Description of Additional Supplementary Files

File name: Supplementary Movie 1

Description: Live cell imaging of the wound healing assay of shControl PC-3 cells over a period of 27 hours. Scale bar, 600 µm.

File name: Supplementary Movie 2

Description: Live cell imaging of the wound healing assay of shCdGAP PC-3 cells over a period of 27 hours. Scale bar, 600 µm.

File name: Supplementary Movie 3

Description: Live cell imaging of the wound healing assay of shControl 22Rv1 cells over a period of 27 hours. Scale bar, 600 µm.

File name: Supplementary Movie 4

Description: Live cell imaging of the wound healing assay of shCdGAP 22Rv1 cells over a period of 27 hours. Scale bar, 600 µm.

File name: Supplementary Data 1

Description: List of differentially expressed genes, 1384 upregulated and 720 downregulated, between shCdGAP PC-3 and shControl cells.
