# Supplementary material for: CdGAP promotes prostate cancer metastasis by regulating epithelial-to-mesenchymal transition, cell cycle progression, and apoptosis
Source: Commun Biol. 2021 Sep 7;4:1042. doi: 10.1038/s42003-021-02520-4 (PMC8423782; doi:10.1038/s42003-021-02520-4)
Supplement: Supplementary file 9 — Reporting Summary [file 42003_2021_2520_MOESM9_ESM.pdf]

nature research

Corresponding author(s): D.P. Labbé and N. Lamarche-Vane

Last updated by author(s): June 28, 2021

Reporting Summary

Nature Research wishes to improve the reproducibility of the work that we publish. This form provides structure for consistency and transparency in reporting. For further information on Nature Research policies, see our Editorial Policies and the Editorial Policy Checklist.

Statistics

For all statistical analyses, confirm that the following items are present in the figure legend, table legend, main text, or Methods section.

n/a	Confirmed	
☐	☒	The exact sample size (n) for each experimental group/condition, given as a discrete number and unit of measurement
☒	☐	A statement on whether measurements were taken from distinct samples or whether the same sample was measured repeatedly
☐	☒	The statistical test(s) used AND whether they are one- or two-sided<br>*Only common tests should be described solely by name; describe more complex techniques in the Methods section.*
☐	☒	A description of all covariates tested
☐	☒	A description of any assumptions or corrections, such as tests of normality and adjustment for multiple comparisons
☐	☒	A full description of the statistical parameters including central tendency (e.g. means) or other basic estimates (e.g. regression coefficient) AND variation (e.g. standard deviation) or associated estimates of uncertainty (e.g. confidence intervals)
☐	☒	For null hypothesis testing, the test statistic (e.g. F, t, r) with confidence intervals, effect sizes, degrees of freedom and P value noted<br>*Give P values as exact values whenever suitable.*
☒	☐	For Bayesian analysis, information on the choice of priors and Markov chain Monte Carlo settings
☒	☐	For hierarchical and complex designs, identification of the appropriate level for tests and full reporting of outcomes
☒	☐	Estimates of effect sizes (e.g. Cohen's d, Pearson's r), indicating how they were calculated

Our web collection on statistics for biologists contains articles on many of the points above.

Software and code

Policy information about availability of computer code

Data collection	no software was used.
Data analysis	no sofware was used.

For manuscripts utilizing custom algorithms or software that are central to the research but not yet described in published literature, software must be made available to editors and reviewers. We strongly encourage code deposition in a community repository (e.g. GitHub). See the Nature Research guidelines for submitting code & software for further information.

Data

Policy information about availability of data

All manuscripts must include a data availability statement. This statement should provide the following information, where applicable:

- Accession codes, unique identifiers, or web links for publicly available datasets
- A list of figures that have associated raw data
- A description of any restrictions on data availability

Data availability
Data are available from the corresponding authors upon request. The sequencing data reported in this paper (RNA-seq) were deposited on NCBI Gene Expression Omnibus (GEO; accession number GSE160399).

Field-specific reporting

Please select the one below that is the best fit for your research. If you are not sure, read the appropriate sections before making your selection.

- [x] Life sciences
- [ ] Behavioural & social sciences
- [ ] Ecological, evolutionary & environmental sciences

For a reference copy of the document with all sections, see nature.com/documents/nr-reporting-summary-flat.pdf

Life sciences study design

All studies must disclose on these points even when the disclosure is negative.

Sample size	Statistical analyses in Figs. 2, 3, 4, 5e,f, 6, 7, 8a-c, 8f, supplementary Figs. 2-4 were performed using GraphPad Prism 9 (GraphPad software, San Diego, CA, USA). A two-sample unpaired Student's t-test was used for comparisons between two groups. Data are presented as the mean +/- SEM and a p-value of less than 0.05 was statistically significant. Data are representative of at least three independent experiments.
Data exclusions	no data were excluded.
Replication	Data are representative of at least three independent experiments.
Randomization	this is not relevant to the study.
Blinding	This is not relevant to the study.

Reporting for specific materials, systems and methods

We require information from authors about some types of materials, experimental systems and methods used in many studies. Here, indicate whether each material, system or method listed is relevant to your study. If you are not sure if a list item applies to your research, read the appropriate section before selecting a response.

Materials & experimental systems

n/a	Involved in the study	
[ ]	[x]	Antibodies
[ ]	[x]	Eukaryotic cell lines
[x]	[ ]	Palaeontology and archaeology
[ ]	[x]	Animals and other organisms
[ ]	[x]	Human research participants
[x]	[ ]	Clinical data
[x]	[ ]	Dual use research of concern

Methods

n/a	Involved in the study	
[x]	[ ]	ChIP-seq
[ ]	[x]	Flow cytometry
[x]	[ ]	MRI-based neuroimaging

Antibodies

Antibodies used	Supplementary Table 3.
Validation	All antibodies used in this study are commercial and have been validated in previous publications.

Eukaryotic cell lines

Policy information about cell lines

Cell line source(s)	ATCC
Authentication	none of the cell line has been authenticated.
Mycoplasma contamination	all cell lines were tested negative for mycoplasma contamination using a PCR test.
Commonly misidentified lines (See ICLAC register)	no commonly misidentified lines used in this study.

Animals and other organisms

Policy information about studies involving animals; ARRIVE guidelines recommended for reporting animal research

Laboratory animals	7-week-old male athymic mice from Charles River.

Wild animals	The study did not involve wild animals.
Field-collected samples	The study did not involve field-collected samples.
Ethics oversight	All animal protocols were approved by McGill University Animal Use and Care Committee, in accordance with guidelines established by the Canadian Council on Animal Care.

Note that full information on the approval of the study protocol must also be provided in the manuscript.

Human research participants

Policy information about studies involving human research participants

Population characteristics	The TMA TF123 series is composed of 300 radical prostatectomy specimens from patients participating in the Centre de recherche du Centre hospitalier de l'Université de Montréal (CRCHUM) prostate cancer biobank.
Recruitment	These patients have undergone surgery at the CHUM between 1993 and 2006.
Ethics oversight	CRCHUM

Note that full information on the approval of the study protocol must also be provided in the manuscript.

Flow Cytometry

Plots

Confirm that:

- [ ] The axis labels state the marker and fluorochrome used (e.g. CD4-FITC).
- [ ] The axis scales are clearly visible. Include numbers along axes only for bottom left plot of group (a 'group' is an analysis of identical markers).
- [ ] All plots are contour plots with outliers or pseudocolor plots.
- [x] A numerical value for number of cells or percentage (with statistics) is provided.

Methodology

Sample preparation	Control or shCdGAP PC-3 cells were serum starved overnight followed by a 24-hour incubation in RPMI containing 10% FBS. 1 x 106 cells were harvested, counted, and washed twice in ice-cold PBS and fixed in 70% ethanol for 1 hour at 4°C. Cells were then washed with PBS and incubated with RNase A for 1 hour at 37°C in a humidified incubator. Finally, cells were stained with 10 µg/ml propidium iodide (PI; Sigma: P4170).
Instrument	BD FACSCanto II system.
Software	FlowJo analysis software v10.7.1 (TreeStar, Inc.)
Cell population abundance	To determine the percentage of cell population distribution, we quantified the population of apoptotic cells with fluorescence in the green emission spectrum (520nm), necrotic cells with red fluorescence (620 nm), and late apoptotic cells with both green and red fluorescence. Data were analyzed using the FlowJo analysis software v10.7.1 (TreeStar, Inc.).
Gating strategy	*Describe the gating strategy used for all relevant experiments, specifying the preliminary FSC/SSC gates of the starting cell population, indicating where boundaries between "positive" and "negative" staining cell populations are defined.*

- [ ] Tick this box to confirm that a figure exemplifying the gating strategy is provided in the Supplementary Information.
